# Ethical guidance for conducting health research with online communities: A scoping review of existing guidance

**DOI:** 10.1371/journal.pone.0302924

**Published:** 2024-05-17

**Authors:** Jane Harris, Jennifer Germain, Ellie McCoy, Rosemary Schofield

**Affiliations:** Public Health Institute, Liverpool John Moores University, Liverpool, United Kingdom; BP Koirala Institute of Health Sciences, NEPAL

## Abstract

Online research methods have grown in popularity due in part to the globalised and far-reaching nature of the internet but also linked to the Covid-19 pandemic whereby restrictions to travel and face to face contact necessitated a shift in methods of research recruitment and data collection. Ethical guidance exists to support researchers in conducting online research, however this is lacking within health fields. This scoping review aims to synthesise formal ethical guidance for applying online methods within health research as well as provide examples of where guidance has been used. A systematic search of literature was conducted, restricted to English language records between 2013 and 2022. Eligibility focused on whether the records were providing ethical guidance or recommendations, were situated or relevant to health disciplines, and involved the use or discussion of online research methods. Following exclusion of ineligible records and duplicate removal, three organisational ethical guidance and 24 research papers were charted and thematically analysed. Four key themes were identified within the guidance documents, 1) consent, 2) confidentiality and privacy, 3) protecting participants from harm and 4) protecting researchers from harm with the research papers describing additional context and understanding around these issues. The review identified that there are currently no specific guidelines aimed at health researchers, with the most cited guidance coming from broader methodological perspectives and disciplines or auxiliary fields. All guidance discussed each of the four key themes within the wider context of sensitive topics and vulnerable populations, areas and issues which are often prominent within health research thus highlighting the need for unifying guidance specific for health researchers. Further research should aim to understand better how online health studies apply ethical principles, to support in informing gaps across both research and guidance.

## Introduction

Globally, there are 5.3 billion and 4.95 billion users of the internet and social media respectively [1], with these online spaces creating unprecedented research opportunities, particularly within health research and leading the internet to be coined ‘the laboratory for the social sciences’ [2,3]. Online methods are frequently used in health research to engage with populations who are hard to reach or seldom heard outside of online spaces, as well as those who are engaging in illicit or risky behaviours [4]. More recently, the Covid-19 pandemic restrictions necessitated a shift in methods of data collection and heightened interest in online methods [5,6]. Online research methods can range from recruiting participants online, e.g., advertising surveys online, through to online communities and social media becoming sources of data through data capture or scraping. It is this latter category where data is ‘taken’ from existing online spaces which often poses the most ethical questions, challenges and blurring of boundaries for researchers [7] who are working in spaces which are changing and adapting all the time as technology advances.

Ethical guidance exists to support researchers conducting online research and comes from both discipline specific fields such as psychology [8] and sociology [9,10], as well as more general guidance from cross disciplinary organisations [11]. This guidance applies offline ethical considerations to online methods, considering issues regarding consent, confidentiality and anonymity as well as protecting both participants and researchers from harm. Whilst these are standard ethical principles, their application can differ in online spaces and guidelines can often be interpreted differently or overlooked entirely within research and by researchers [12]. Furthermore, the study of online communities can present new ethical considerations such as whether these spaces can be considered public or private, a distinction which is difficult to navigate within the context of online platforms and where views are inconsistent even by those who inhabit such spaces [13]. Whilst online research has been used extensively within social sciences and particularly within health research, no guidance exists which is aimed specifically at health researchers. This coupled with complex ethical issues within an ever-changing online landscape can make conducting this research challenging. Therefore, this scoping review aims to synthesise current best ethical practice and guidance for online research and specifically for health researchers, consider the application of these sets of guidance within health research.

## Methods

Due to a paucity in guidance specifically aimed at health researchers, this scoping review aimed to synthesise existing formal online ethical guidance within health research. Arksey and O’Malley’s [14] five-stage iterative process for scoping reviews was followed throughout, comprising the following 5 stages: (1) identifying the research question, (2) identifying relevant studies, (3) study selection, (4) charting the data and (5) collating, summarizing and reporting the results.

### Research question

The review aimed to understand what ethical guidance is available for researchers conducting online research. The underpinning research question was *‘what guidance exists on the ethical considerations that should be taken by health researchers conducting research with or about online health communities’* which guided the systematic search strategy. The overarching review question allowed the reviewers to search a wide range of available literature to capture what guidance is already available in the public domain and collate and review recommendations developed from other studies.

Aims and objectives

Specifically, this study aimed to:

- Map the available guidance on online ethics that is of relevance to health researchers
- Thematically describe the key ethical considerations covered by this guidance (e.g., consent, protection from harm, preventing identification, verbatim quotes)
- Highlight any areas of disagreement across the guidance
- Identify any gaps or ethical grey areas in current guidance.

### Identifying relevant studies

The search strategy was developed using search terms based on the PCC framework [15,16]; population of interest (health researchers), the concept (ethical guidance and considerations) and context (research with online health communities). JH and JG led the development of the search strategy and search terms focused on 1) online 2) research 3) ethics and 4) policy and guidance. Searches were conducted across Scopus, Web of Science, Medline and Psycinfo to locate publications over a 10-year timeframe between 2013 and 2022. A ten year timeframe was chosen due to rapidly evolving trends across social media and technology, with this time frame best reflecting the current online platform landscape. Manual searching of reference lists was undertaken and grey literature searched using Google Scholar, Google and websites of key health related research bodies and regulators (e.g., NIHR, BPS, Royal College of Physicians).

Searches identified 3,294 records which were imported into Rayan online reference manager and duplicates removed (n = 1,177). Study selection utilised a two-step process to screen titles and abstracts (n = 2,117) and then full paper screening (n = 189). All screening was undertaken by JH, JG, EMC and RS and following inclusion and exclusion criteria set out in Table 1. Two reviewers each blind screened papers at both screening stages with conflicts resolved by group discussion and a third review where necessary. Research papers were selected for the review if they were conducting one of the forms of online research outlined in Table 1 in a health-related field and if their article made recommendations or guidance of their own for ethical practice as a result of their research experiences. Grey literature followed the same two-step process for screening. Twenty-seven articles and guidance were selected for data charting. All searches were conducted between February and June 2022, and were repeated in August 2023 however no new guidance was identified in these latter searches.

**Table 1:** Inclusion/exclusion criteria for study selection.

Time	Only papers published after 31/12/2012
Study design	Article must provide guidance, recommendations or reflect on the methodological issues related to conducting online research in an ethical manner
Population	The guidance/article should be aimed at researchers in a health-related field (or health professionals conducting research) OR The guidance/article must be applicable to researchers in a health-related field as part of a broader target audience (e.g., social science research, qualitative research etc.)
Research method	The guidance/article must focus on one of the following: • Scraping or extracting health or behavioural data from existing online communities (e.g. forums, social media, video sharing sites, blogs) • Using existing online communities to recruit participants to health-related research • Creating online communities (e.g., Facebook groups, message boards) for the creation of health-related research data • Creating online communities for the recruitment of participants to health-related research
Outcomes	The outcomes should be in the form of guidance, policy or recommendations for researchers to improve their ethical practice in relation to online health research

### Risk of bias

Whilst quality assessment is not a requirement of scoping reviews, assessing the methodological and other qualities of studies can support in contextualising findings and enable interpretation [15]. Therefore, the selected articles were assessed for quality and bias using the Joanna Briggs Institute (JBI) Critical Appraisal Checklist for Text and Opinion [17]. The Text and Opinion checklist was considered most suitable as the scoping review had identified a range of different publications which made recommendations on ethical practice in online health research including organisational guidance documents, commentary and opinion pieces and methodological papers. As presented in Table 2, the checklist criteria focused on whether the ethical recommendations made were clearly identified and logical, had standing in their field of expertise, were relevant to health researchers and had congruence with existing research. The quality of the papers and guidance was independently assessed by one of the four reviewers (JH, JG, EMC, RS) using the predetermined questions. The quality assessment of the papers can be viewed in Table 2. The review papers largely met the criteria and after discussion, the decision was made to include all 27 papers in the final review.

**Table 2:** JBI critical appraisal checklist to assess trustworthiness, relevance and results of selected papers.

Author (year)	Is the source of the opinion clearly identified?	Does the source of opinion have standing in the field of expertise?	Are the interests of the relevant population the central focus of opinion?	Is the stated position the result of an analytical process, and is there logic in the opinion expressed?	Is there reference to the extant literature?	Is any congruence with the literature/sources logically defended?	Overall appraisal
Anabo et al (2019) [18]	Yes	Yes	Yes	Yes	Yes	No	Include
Andanda et al (2020) [19]	Yes	Yes	No	Yes	Yes	Yes	Include
Arigo et al (2018) [20]	Yes	Yes	Yes	Yes	Yes	Yes	Include
Azer (2017) [21]	Yes	Yes	Yes	No	Yes	No	Include
Bender et al (2017) [22]	Yes	Yes	Unclear	Yes	Yes	Yes	Include
Benton et al (2017) [23]	Yes	No	Yes	Unclear	Yes	Unclear	Include
BPS (2021) [8]	Yes	Yes	Yes	Yes	Yes	Yes	Include
BSA (2017) [9]	Yes	Yes	Yes	No	Yes	Yes	Include
Buck et al (2021) [24]	Yes	Unclear	No	Yes	Yes	Yes	Include
Burles et al (2018) [25]	Yes	Yes	Yes	Yes	Yes	Yes	Include
Cilliers et al (2020) [26]	Yes	Unclear	Yes	Yes	Yes	Yes	Include
Clark et al (2019) [27]	Yes	Yes	Yes	Yes	Yes	Yes	Include
Curtis (2014) [28]	Yes	Unclear	Yes	Yes	Yes	Yes	Include
DeCamp (2015) [29]	Yes	Unclear	No	Yes	Yes	Yes	Include
Ford et al (2021) [30]	Yes	Yes	No	Yes	Yes	Yes	Include
Franzke et al (2020) [11]	Yes	Yes	Yes	Yes	Yes	Yes	Include
Gelinas et al (2017) [31]	Yes	Yes	Yes	Yes	Yes	Yes	Include
Gupta (2017) [32]	Yes	Unclear	Yes	Unclear	Yes	Yes	Include
Harris et al (2020) [33]	Yes	Yes	Yes	Yes	Yes	Unclear	Include
Hunter et al (2018) [4]	Yes	Yes	Unclear	Unclear	Yes	Yes	Include
Lapadat (2019) [34]	No	Unclear	Yes	Unclear	Yes	Yes	Include
Moreno et al (2016) [35]	Yes	Yes	Yes	Yes	Yes	Yes	Include
Perez-Vallejos (2017) [36]	Yes	Yes	Yes	Yes	Yes	Yes	Include
Sugiura (2017) [10]	Yes	Yes	Yes	Yes	Yes	Yes	Include
Taylor et al (2018) [37]	Yes	Yes	Yes	Yes	Yes	Yes	Include
Warrell (2014) [38]	Yes	Yes	Yes	Yes	Yes	Yes	Include
Yadlin-Segal (2020) [39]	Yes	Yes	Yes	Yes	Yes	Yes	Include

### Data collection, summarisation and presentation of results

A data extraction form was developed in a Microsoft Excel Spreadsheet charting all relevant data including authors, year of publication, key ethical topics covered and, for research papers, which if any ethical organisational guidance had been followed. The data form was piloted independently by two authors (JH/JG) on 10% (n = 3) of papers, and the remainder of included studies split between the two reviewers. Data was charted across four key areas *1) consent*, *2) confidentiality and privacy*, *3) protecting participants from harm and 4) protecting researchers from harm*. The areas where initially informed by the key subheading across all included ethical guidance and refined through discussions involving all members of the team. All data was systematically extracted and charting was conducted by two researchers (JH and JG) and checked for consistency. The researchers then made comparisons across the selected papers to identify common themes and gaps across the existing guidance. Once data extraction was complete, JH and JG discussed the findings to identify the key points of ethical consideration within each theme which were then synthesised to create a narrative account of the guidance.

## Results

**Fig 1:**
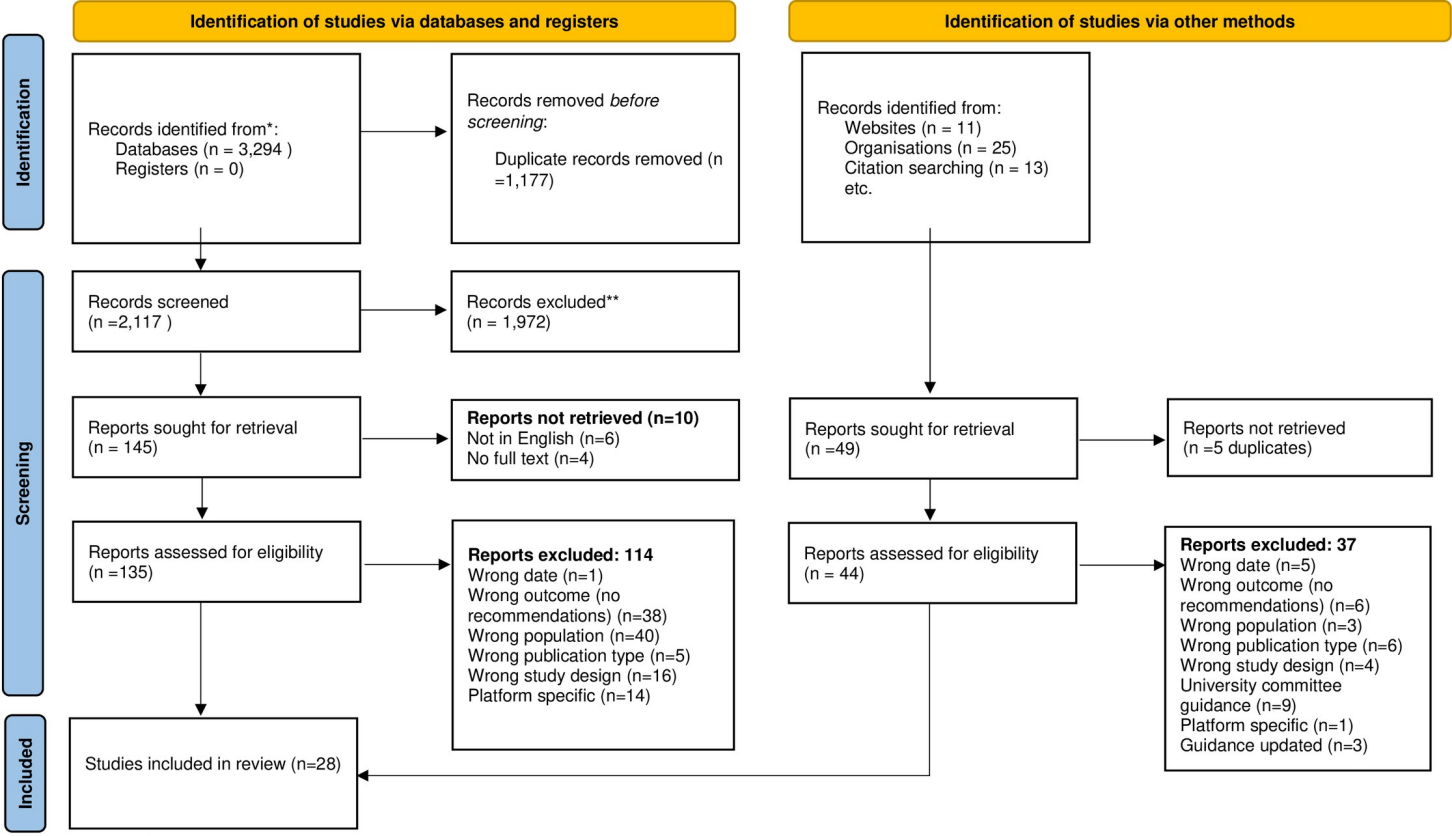
PRISMA flow diagram.

Guidance from three organisations met the inclusion criteria for the review (See Fig 1 for PRISMA flow diagram): the British Psychological Society (BPS) [8], the Association of Internet Research (AoIR) [11] and the British Sociological Association (BSA) [9,10].

**Fig 2:**
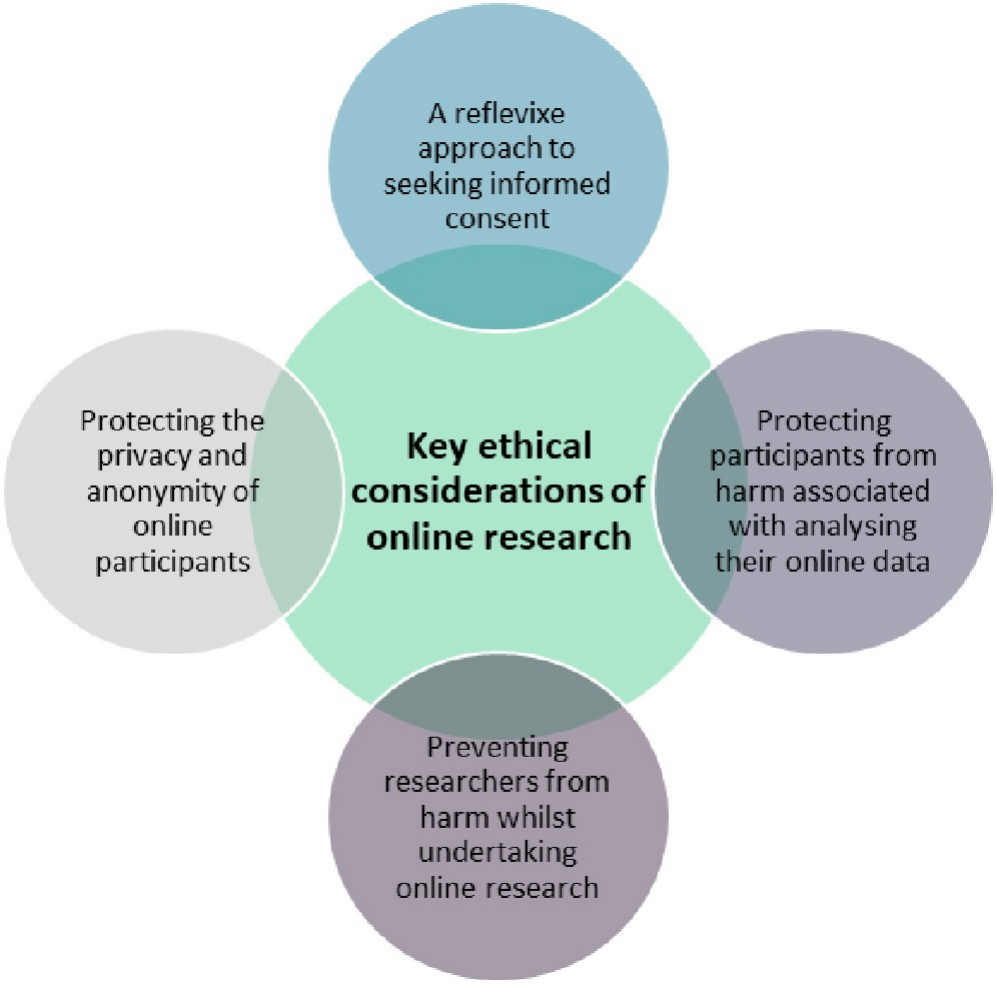
Summary of key themes from guidance on ethical online research.

Summaries of the guidance are included in Table 3 and our narrative is presented around four key themes: consent, confidentiality and privacy, protecting participants from harm and protecting researchers from harm (Fig 2). Alongside these guidance documents, 24 research papers (Table 4) were included to give a clearer understanding of which (if any) ethical guidance health researchers were following. The AoIR [11] was the most commonly cited guidance (n = 18), followed by the BPS [8] (n = 7), with the BSA [9] least cited (n = 1). Six papers did not cite any of the guidance documents, however, most papers were following the key guidance principles.

**Table 3:** Summary of critically appraised online ethics guidance documents.

Author	Organisation Produced on behalf of	Consent	Privacy/anonymity	Protecting participants from harm	Protecting researchers from harm
BPS (2021)[8]	British Psychological Society (BPS) (second edition)	• Valid consent should be obtained where data not reasonably considered in public domain or no scientific justification for undisclosed use. • Difficult to verify participant characteristics (e.g. age) and confirm engagement with consent procedures. • Care not to over complicate processes so that they are read and participants who wish to proceed can do so. Proportionality to level of risk. • Withdrawal–Clear exit/withdrawal button and debrief needed. For unobtrusive approaches withdrawal may be required if participant finds their online posts have been accessed and stored.	• Distinction between public and private is blurred online because communication can take place privately (in home) and publicly (public discussion board) simultaneously. • When there is ambiguity researchers should consider extent potential damaging effects of undisclosed observation before deciding if valid consent needed. Group moderator can often provide advice on best ways to research existing online groups. • Data must remain confidential, and safeguards must be proportional to the risk of potential harm. • Legislative concerns: copyright.	• Steps to maximise benefits and protect participants from harm: gaining valid consent, ensuring anonymity and confidentiality and maintaining appropriate levels of control over research process. • Lack of control can lead to issues in verifying identity and checks should be proportional to the risk for harm. • Research on sensitive topics best avoided if control low and risk high. • Would publishing traceable quotes pose risk and non-trivial harm. Paraphrasing or combining quotes, not publishing name of online community, removing pseudonyms and identifiable information. • Social responsibility to avoid disrupting social structures, trust and cohesion of online groups.	• Scientific value: reduced control particularly in unobtrusive research over who participants, environmental conditions of participation, responses to research process and research procedure on different software/hardware can impact on validity.
franzke et al (2020)[11]	Association of Internet Researchers (AoIR) (third edition)	• Seeking informed consent must consider the online environment where interactions take place and the expectations of both this venue and participants. • Informed consent: specific considerations e.g timing, medium, participants, specific research purposes. • Emerging issues is informed consent in big data projects–strong steps needed to protect individual identity when informed consent is not possible.	• Differing approaches to ethics and privacy in Europe (deontological–prioritise protecting rights of autonomous individuals) and UK & US (utilitarian–greater good for society), so endorse ethical pluralism. • Terms and conditions of social media platforms must be considered. • Data minimisation–only collecting enough for research purposes vs big data which inductively seeks answers from large datasets.	• The greater the vulnerability of participants, the greater responsibility to protect them from likely harms. • Specific considerations include: Downstream harms or harms after the fact, Minors, Politically sensitive research, Special emotional states such as grieving and/or trauma, illnesses; minorities, LGBTQ+ communities. • Disclosure of behaviour threatening to participant well-being, e.g., self-cutting or suggesting the potential for committing crimes. What are the researchers obligations in reporting this to relevant authorities and platforms on which they appear?	• Growing need for protecting the researchers, as well as informants. New risks for researchers whose work–and public identity (e.g., ethnicity, minority identity, sexual identity, political activism, etc.)–triggers strong ideological reaction or research on political extremes: these include death threats, “doxing”. • Exposure to extreme online content can have psychological impact on researchers. Offers resources for enhancing researcher safety and argues institutions should develop policy detailing support procedures for researchers experiencing online threats or harassment related to their work.
BSA (2017) [9] and Suguira (2017) [10]–*case study annex*	British Sociological Association (BSA)	• Consent is a complex issue. While not legally required to access publicly available data, exemptions must be carefully justified. Threats to privacy above those which already exist, data produced by public agencies, when people agree to being identified, when people should be credited as authors, illegal activities, use of recording technology. • Dialogic–two-way ongoing communication between researcher and participant. • If research focuses on large scale data obtaining consent and communication can be challenging or impossible. • Pitfalls both from attempting to obtain informed consent and bypassing it. Obtaining requires joining community, revealing identity and purpose of study which can be precarious if topic is sensitive. Might disrupt naturalistic research environment. Practical difficulties getting consent from all members (not see posts, left forum). • Covert approach could enable research on public sites without risk or harm to community, but must consult terms of forums or contact moderator to gain permission.	• Boundaries of public/private blurred online: many may not expect to be observed leading to a mismatch in expectations of privacy between researcher and participants. • Researchers can familiarise themselves with the site to ascertain whether it should be considered public from the perspective of those who occupy it. • Protect privacy through anonymization removing sensitive information for example about personal illness. Verbatim quotes should not be published as those direct quotes can be searched. Undecided whether full quotes need permission, though would suggest this is likely the case.	• Underlying principle should be care of our participants Must maximise the benefit and minimise the harm through values of protection, respect, dignity, and privacy. Situational ethics rather than absolutes or right or wrong. • Existing online data–no control over how collected, consent principles cannot be readily applied at scale. • May raise new ethical challenges e.g. linking data about individual from multiple sources.	• Must always secure institutional ethics approval. Where situational ethics are applied these should be the subject of documentation and report, if necessary to the appropriate ethics committees. • Working online and with new forms of data, may place researchers in vulnerable positions, making them publicly visible and at risk of abuse and steps should be taken to protect researchers.

**Table 4:** Summary of critically appraised selected papers which have used and made recommendations on online research ethics.

Author (year)	Guidance Stated	Consent	Privacy/anonymity	Preventing from Harm
Anabo et al (2019)[18]	BPS, BSA, AoIR	• BPS: scientific and social value outweighs risk. • AoIR, BSA: consent is an ongoing process.	• BPS: paraphrasing. Anonymity cannot be guaranteed due to text archiving web services.	• AoIR (participants)—proportionality of harm according to vulnerability of group. Vulnerability from study can’t always be predicted and should be reflexive and negotiated in context. • BSA (researchers) wary of putting selves in position of harm and online abuse.
Andanda (2020) [19]	AoIR	• BPS (not referenced): consent should be sought when no scientific justification for undisclosed use. • AoIR, BSA: need to consider environmental context and need for ongoing participant communication.	• BPS, BSA (not referenced): anonymisation of data needed including online pseudonyms.	• AoIR (referenced), BPS and BSA (not referenced): consideration of potential harm should be sensitive to the context.
Arigo et al (2018) [20]	None	n/a	n/a	n/a
Azer (2017) [21]	None	• More extreme view than BPS saying online spaces should always be considered private and consent should always be sought.	• BPS and BSA (not referenced): that participants may have varying perceptions of privacy and not expect their data to be used for research purposes.	• BPS, BSA, AoIR (not referenced): identification of potential harm and prevention is context specific.
Bender et al (2017) [22]	AoIR	• More strict than guidance—consent should always be sought.	• BPS (not referenced): balance between effective communication of consent and privacy and not making the process so complicated that it prevents individuals from participating.	• BSA, BPS, AoIR (not referenced): agree harm is contextual and certain groups may be at greater risk.
Benton et al (2017) [23]	None	• BPS (not referenced): consent should be sought if not in public domain or scientific justification. • AoIR, BSA (not referenced): Challenge for big data studies. Additionally, where consent not sought that a "statement of responsibility" should be posted on the research groups website stating how, what, why data sought and how protected—suggest people could thus opt out if they access this.	• BPS, BSA (not referenced): blurred boundaries and expectations of privacy in online spaced. • BPS, BSA, AoIR (not referenced): Practical steps of anonymisation, removing sensitive info and paraphrasing or combining quotes Specifically creating synthetic quotes but must clearly state to reader when quotes are artificial.	• BPS, BSA, AoIR (not referenced): steps such as de-identification should be proportional to the risk of harm. User perceptions of what is sensitive. • Caution about linking data across multiple platforms as this can reveal more about individual’s online persona and could "out" sensitive info such as health condition.
Buck and Ralston (2021) [24]	AoIR, BPS	• AoIR, BPS: studies of discourse may not require consent whilst studies of individuals will. Difficulties of confirming digital consent is informed even when sought.	• AoIR and BPS: blurred expectations privacy among online users. • BPS: Recommend anonymisation and paraphrasing. • AoIR; recommend considering legal and platform regulations.	• BPS, AoIR: context is important when considering harm. • Highlight considering whether your online participants/group is representative of the community of study. • AoIR (referenced) and BSA (not referenced): researchers could be at increased risk of online abuse, particularly more vulnerable groups including women and minority groups.
Burles et al (2018) [25]	AoIR	• AoIR (referenced), BPS (not referenced): in some cases, the need for consent is clear (e.g. generating online data specifically for study or in private spaces) but in other public spaces it is more ambiguous. • AoIR: contextual factors and community expectations are important • BSA (not referenced). • BSA, BPS (not referenced): Must not disrupt the online community through obtrusive research practices.	• AoIR (referenced), BPS (not referenced) Consider steps such as anonymisation, paraphrasing or fabrication to prevent identification.	• AoIR(referenced), BPS (not referenced) maximising benefit and minimising harm. Context responsive particularly for sensitive groups.
Cilliers and Viljoen (2020) [26]	AoIR	n/a	• AoIR (referenced), BPS (not referenced): expectations of privacy blurred online (AoIR, BPS—not referenced). • AoIR (referenced), BPS, BSA (not referenced): issues of reidentification (even from large datasets. Anonymisation and paraphrasing.	• BPS (not referenced): that should consideration of harm should be contextual. • This paper suggests research with sensitive populations should only be undertaken where it is not possible to do this with non-sensitive population. • BPS (not referenced): research integrity through identifying participant characteristics. Age verification and inability to gain parental consent.
Clark et al (2019) [27]	AoIR	• AoIR (referenced), BPS (not referenced) online consent—ensuring information is read. Can be impractical with large amounts of public data. • This paper suggests communicating research purpose prior and providing a debrief. • Additional issue highlighted—prospective studies (e.g., using Twitter data to predict post-natal depression)—collecting data which could be sensitive but not yet produced, how manage consent?	• AoIR (referenced) and BPS (not referenced): public data and use of data for research not always expected by posters. • AoIR (referenced) BPS, BSA (not referenced): depends on sensitivity and vulnerability of population. • AoIR (referenced) different cultural understandings and legislation around privacy. • AoIR (referenced), BPS, BSA (not referenced): deidentification of data.	
Curtis (2014) [28]	AoIR	• AoIR (referenced), BPS, BSA (not referenced): challenges of age verification and assessing comprehension of consent. • This paper suggests clear summaries and FAQs, seeking feedback on consent process and using an online quiz to check study comprehension.	• AoIR (referenced), BPS, BSA (not referenced): highlights particular issues for vulnerable groups. Suggests anonymisation (BPS—not referenced), not naming sites (BPS—not referenced) and removing and sensitive health information (BSA—not referenced).	
DeCamp (2020) [29]	None	• BPS (not referenced) need to verify characteristics. And inability to gauge understanding of information. • This paper suggests using a test or quiz to confirm understanding.	• AoIR, BPS, BSA (not referenced) privacy is blurred in online spaces. • BSA and BPS (not referenced) familiarisation with sites and discussions with form moderators: 1) understand site expectations and T&Cs 2) if registration and approval of moderator required, there is some expectation of privacy 3) site organised around sharing stories there might be expectation of privacy and researcher should engage with users and moderators before beginning research.	
Ford et al (2021) [30]	AoIR	• AoIR (referenced), BPS, BSA (not referenced): consent decisions should b made in relation to expectation of privacy and risk of disclosure. • This paper suggests consent needed were login required to access data as an expectation of privacy.	• AoIR (referenced), BPS, BSA (not referenced) blurred privacy expectations online. • AoIR (referenced), BPS, BSA (not referenced) anonymity through removal of personal information, paraphrasing or combining quotes. • AoIR (referenced): preserve anonymity of vulnerable groups.	• AoIR (referenced), BPS (not referenced) challenge of predicting harms. Must consider vulnerable groups, sensitive topics, young people. • AoIR (referenced), BSA (not referenced): seeking ethical approval to protect researchers.
Gelinas et al (2017) [31]	None	• n/a	• AoIR, BPS, BSA (not referenced): blurred interpretations of privacy online. • BPS, BSA (not referenced): protect participants from identification particularly in relation to sensitive information such as health information BPS, BSA (not referenced): consulting with forum moderators and users to understand their expectations of privacy.	• n/a
Gupta (2017) [32]	AoIR	• AoIR (referenced) and BPS, BSA (not referenced): recommend gaining informed consent. • AoIR (referenced), BPS (not referenced) However difficult to verify online if this is read and participant characteristics. • This paper suggests multi-stage consent forms.	• AoIR (referenced) and BPS, BSA (not referenced) suggest that online users expectations of privacy must be considered. • AoIR (referenced), BPS, BSA (not referenced): recommend removing all identifiers before analysis. • This paper recommends inclusion of a privacy statement.	• BPS, BSA (not referenced): maximising benefits and minimising harm—confidentiality must be considered proportionally to the level of harm.
Harris et al (2020) [33]	AoIR, BPS	• BPS: public domain data and scientific justification with low risk of harm then consent may not be required. • BSA (not referenced): seeking consent from forum moderators. • BPS: Challenges of validation of demographic information.	• AoIR, BPS (referenced), BSA (not referenced): blurred public and private boundaries. must respect community desires for privacy and this can be sought via moderators. • BPS, AoIR: Anonymising of data and google proofing of quotes relative to privacy—paraphrasing more important from public online site than private.	• BPS, AoIR: anonymity. Must have a clear understanding of the community being researched. Consider site netiquette and any potential risks to do with cohesion and trust. • AoIR (referenced), BSA (not referenced): protecting researcher harm by seeking ethical approval.
Hunter et al (2018) [4]	AoIR, BPS	• AoIR, BPS (referenced), BSA (not referenced): users often consent to online social media without being fully aware what they are consenting to therefore implied consent should not be the default position. • Withdrawal challenging (e.g., when people delete data).	• AoIR, BPS: Varying perceptions of privacy. Recommend anonymising data including google-proofing	• BPS, AoIR: Increased risk when researching sensitive topic or vulnerable communities. Clear protocols for how emotional distress will be handled • AoIR: risk of emotional impacts when exposed to sensitive or politically extreme online content. Clear descriptions of researchers’ roles and debriefs should be available
Lapadat (2019) [34]	AoIR	• n/a	• AoIR (referenced): that privacy laws vary across the world and researchers must be responsive to this as well as individual expectations.	• AoIR (referenced), BPS (not referenced): inability to verify certain characteristics such as age or vulnerable population increases risk of harm and should be taken into account. • AoIR: blanket restrictions on access to API on Twitter could lead to reduction in meaningful research over time.
Moreno et al (2016) [35]	None	• AoIR, BPS, BSA (not referenced): complexities of consent, researchers should consider seeking consent if verbatim quotes are used. • AoIR, BPS, BSA (not referenced): issues of seeking parental consent and agree this should be relative to the sensitivity of the topic.	• BSA and BPS (not referenced) steps to reduce the risk of participant identification through anonymisation and avoiding direct quotes. • This paper recommends listing privacy procedures for research publicly on lab/research group page.	• n/a
Perez Vallejos et al (2017) [36]	AoIR and BPS	• BPS: covert research without informed consent must have scientific justification and ethical approval must be sought. Suggest seeking of gatekeeper consent (e.g. forum moderators). • BPS: acknowledge difficulties of informed consent online and balance is needed between relevant information and not overly long or complicated consent processes. • This paper also suggests ensuring opportunity for participants to make enquiries, withdraw and debrief. Identifying those who cannot give informed consent is more challenging online and suggest consulting with subject/clinical experts as relevant for guidance.	• BPS (referenced), BSA (not referenced): researcher responsibility to ensure confidentiality even if not online participants expectation through anonymisation, data minimisation and removal of personally identifiable data from stored datasets—including geolocation data.	• BPS (referenced), BSA (not referenced): Participants’ risk of emotional harm through breach of privacy or sensitive topics and wider social harm. • BPS, AoIR (referenced), BSA (not referenced)L Protect researchers from harm through familiarisation with ethical protocols, seeking ethical approval and identifying potential harms.
Suguira et al (2017) [40]	AoIR, BPS, BSA	• BPS: consent where data is not in public domain or no scientific justification for use.	• BSA, AoIR, BPS: reduce risk of identification through removal of personally identifiable. • AoIR, BPS, BSA: paraphrasing or condensing of verbatim quotes.	• BPS, AoIR, BSA: proportional to the risk of harm.
Taylor et al (2018) [37]	BPS, AoIR	• n/a	• n/a	• n/a
Warrell and Jacobson (2014) [38]	AoIR	• Simply state that information publicly posted online and accessible to anyone is open to being included in research data. • AOIR (referenced), BPS, BSA (not referenced): non-physical presence of participants in the virtual world also makes it difficult for researchers to verify certain information including age and capacity to consent.	• AoIR guidelines asked researchers to consider whether they view their participants as hu-man subjects or as authors (Ess & Association of Internet Researchers, 2002). If viewed more as an author, the researcher may lean towards giving credit where credit is due. If, however, the participant is viewed as a human subject to be protected, the researcher is likely to ensure the individual’s anonymity is maintained. Anonymous participation is part of the attempt to minimize any harms or risks to reputation, professional standing, or other personal or professional matters that may result from that person being associated with the research or its findings. However, if the participant is considered an author, anonymity deprives the contributor of credit for their work.	• n/a
Yadlin et al (2019) [39]	AoIR	• AoIR: consent should be an inductive process. • BPS, BSA (not referenced): seeking moderator consent as gatekeepers.	• AoIR (referenced) BPS, BSA (not referenced) blurred lines of public/private online. However, authors argue lurking is an ethical issue related to privacy. • AoIR (referenced), BPS, BSA (not referenced) anonymisation and paraphrasing of quotes.	n/a

### Consent

All guidance discussed the complexities of obtaining informed participant consent in online research with BSA [9] and AOIR [11] noting particular difficulties for big data studies due to the sheer number of participants involved. According to the BSA, whilst informed consent is not legally required to obtain data from public online spaces, it cannot be overlooked from an ethical perspective [9,10], particularly when topics are sensitive in nature [10]. The BPS [8] assert that if data is not considered in the public domain and there is no scientific justification for undisclosed use, consent should always be gained. The AOIR [11] state that consent should consider environmental factors and expectations of the online communities and users under investigation. A number of practical difficulties were noted in obtaining consent including users having left online spaces and the potential disruption to online communities [10]. The BPS [8] outline the need to verify participant characteristics including vulnerability to coercion and ensure consent processes are not overly complicated and lengthy. BSA [9] advocate for a participatory approach of ongoing communication and recommend researchers consult forum moderators for permission (although they acknowledge moderators may not speak for all users) [10].

The majority of papers (n = 13) concurred that consent was more ambiguous in public online spaces [8,11] and was an ongoing process requiring continual reflection and communication with participants [4,18,19,23,25,27,30,32,33,35,36,38–40]. Eight papers repeated the BPS assertion that not seeking consent for public domain online data could be justified when the scientific and social value outweighed the associated risk [18,19,23,30,33,36,38,40]. Examples included data on non-sensitive health topics taken from large publicly accessible forums (for example Mumsnet) and blog or vlog data produced for a public audience [25,27,33]. Two studies [21,22] took a more extreme view that all online spaces are private, and consent should always be sought. However, in these instances both were discussing recruiting participants from online spaces to take part in conventional research.

In line with AoIR, seven papers gave attention to the expectations of online communities in relation to research [19,25,30,33,36,39,40]. For example, Burles et al [25] suggested that disrupting online illness support communities could cause members to change or reduce the support they provide to others. Three papers agreed with the BSA that consent should be sought from community moderators [40] using the examples of a private fathering forum [33], an online youth counselling platform [36] and closed Facebook groups [33,39]. However, seven papers acknowledged challenges of verifying age characteristics online [26,28,29,32,34–36] and the ability to give or capture informed consent online [36], and recommended consent processes relative to topic sensitivity [35] developed in consultation with clinical and subject experts [36]. Five papers acknowledged the challenges of ensuring participant information was read by participants [27–29,32,36] with solutions including FAQs [28], online quizzes [28,29] and multi-stage consent forms [32]. The scale of online datasets was also viewed as a barrier to seeking individual level consent [8,9,11]. Solutions proposed included seeking consent for quotes used in publications [11,27], as well as the posting of debriefing messages on the online communities or a dedicated study site [27,36,40]. Finally, three papers discussed the challenges of withdrawing consent in online research, for example when data included in a study is deleted by the user [4,36] or in prospective studies when the sensitive data has not yet been produced, with Clark et al [27] giving the example of using Twitter posts to predict post-natal depression.

### Confidentiality/Privacy/Anonymity

As the guidance on consent indicates, all three guidance documents acknowledge that privacy is challenging to define in online spaces [8,9,11]. The BPS generally defines a public space as one where participants could reasonably be expected to be observed by strangers [8]. However, their internet mediated research guidance acknowledges this distinction is not so clear cut for online research because online interaction can simultaneously take place publicly (for example a public online forum) and privately (from the home) [8]. Where this ambiguity exists the BPS [8] and BSA [9] propose several practical steps to ensure participants are sufficiently protected from harm which are discussed in the section below. As the only international guidance included, the AoIR [11] gives a wider contextual outlook at differing perspectives between disciplines and across countries. They highlight the difference between deontological approaches to privacy (protecting the rights of autonomous individuals) in Europe versus the more utilitarian approaches (achieving a greater good for society) taken in the UK and US and advocate for “*ethical pluralism”* which acknowledges these legitimate differences but develops shared norms and practices. The AoIR highlights several contextual factors which must be considered alongside the practical steps outlined by the BPS [8] and BSA [9]. This includes legal frameworks (e.g., GDPR), platform terms and conditions, management of data, adequate anonymisation of large data sets and reliance on companies giving access to their API which can favour certain countries, universities and researchers.

Twelve papers reflected on the blurred lines between public and private spaces online and users varying expectations of privacy in relation to research [4,21,23–27,29–33,36]. For example DeCamp [29] made recommendations which aligned well with the guidance, stating: 1) researchers should understand site Terms and Conditions regarding privacy and research participation, 2) if a site requires registration and moderator approval, there is some expectation of privacy, and 3) sites with express purpose of sharing individual health stories might have some expectation of privacy and researchers should engage with moderators and users before beginning the research. Two papers recommend the publication of a privacy statement via study or research group specific websites [18,32,35]. The issues highlighted by the AoIR in relation to different legal and cultural understandings of privacy internationally were rarely discussed, with only three papers providing any reflection on this [26–28].

### Participant harm

The BSA [9] and BPS [8] both argue that online research should maximise benefits and minimise harm through values of protection, respect, dignity, and privacy [9] and gaining informed consent and anonymisation are vital to this. The BPS highlight social responsibility to respect the social structures of existing online groups and the consequences of undertaking research upon group cohesion and trust. Several practical steps are recommended to address this. These include: familiarisation with the online space of study to ascertain if participants perceive it to be public [9], discussions with group moderators on the best way to research their online groups [8,9], anonymisation of data through removal of personal information [8,9] including online pseudonyms [8], not including potentially embarrassing or sensitive information [9], paraphrasing or combining quotes [8,9], age verification and not naming online communities [8]. The guidance also note that these actions should be proportional to the risk of harm, for example sensitive topics and more vulnerable groups including children and young people, women, certain emotional states such as grieving and/or trauma, illnesses; and minorities such LGBTQ+ communities [8,11]. Research on sensitive topics where risks are high and the ability to control is low should be avoided and consideration must be given to researchers’ responsibility in reporting online users discussing negative well-being and criminal activity [8].

Sixteen of the studies recommended the anonymisation of data [4,19,23–28,30–33,35–37,40], achieved through removing online pseudonyms, sensitive health information, geolocation data and the names of sites from which data was collected. Benton et al [23] advise caution when linking data across multiple platforms as individual online personas could “out” sensitive health information. Ten studies recommended paraphrasing verbatim quotes [4,18,23–26,30,33,35], and this varied from changing minor words to prevent retrieval form a search engine, through to the fabrication of synthetic quotes based on participants’ words [23,25]. Eighteen papers felt potential harm was dependent on the sensitivity and anonymity of each online group and that vulnerability should be sensitively negotiated as the research progresses [4,18,19,21–24,26–32,34–36]. Three papers suggest this is addressed through familiarisation with site netiquette and potential impacts on cohesion and trust [29,31,33].

### Researcher harm

Two of the three guidance discussed protecting researchers from harm [9,11]. This was considered particularly important for research relating to politically sensitive topics or where researchers hold identities (e.g., ethnicity, minority identity, sexual identity, political activism, etc.) that could trigger strong ideological reactions [11], placing them in publicly visible, vulnerable positions and at risk of abuse [9]. Both guidelines called for the support from individual institutions, stating that researchers should receive ethical approval prior to commencing research and continue to discuss challenges with the committee throughout [9]. Furthermore, institutions should develop policy detailing support procedures for researchers experiencing online threats or harassment related to their work [11]. The BPS [8] also highlight the challenges of ensuring integrity and scientific value when researchers have less control over who can participate, environmental conditions and responses during the research and variations in the research procedures caused by different hardware and software.

Seven papers considered the potential researcher harms from online research. Two papers noted that researchers should be cautious of putting themselves at risk of online abuse particularly if they were from groups vulnerable to harm [18,24]. Hunter et al [4], in line with AoIR [11], discuss the emotional impacts of exposure to sensitive or politically extreme information online, recommending clearly defined researcher roles and regular debriefs when exposed to content. Five papers reflect on the impacts upon research integrity raised by the BPS [8,26,30,33,36] with three papers suggesting that seeking approval from institutional research ethics committees is the best way to ensure methodologically and ethically sound research [30,33,36].

## Discussion

Our scoping review identified three online ethical guidance documents published within the last ten years which were relevant to researchers working across health-related disciplines: the Association of Internet Researchers (AoIR) [11], the British Psychological Society (BPS) [8], and the British Sociological Association (BSA) [9]. Whilst our review identified common thematic components across this guidance, it is also important to acknowledge that these guidance documents differ in scope and intention. The AoIR guidance promotes ethical pluralism, by highlighting key principles which are relevant to multidisciplinary researchers globally. In contrast, the BPS and BSA are country and discipline specific, presenting structured guidance on research practices. Our review identified a notable gap in formal guidance produced by international and national health organisations. As a result, a number of papers were identified which aimed to give advice to health researchers on conducting ethical online research and these papers drew on a combination of the existing guidance from other disciplines and research experience. Seventeen of the selected papers cited at least one of the three guidance documents, highlighting a clear will among health researchers to make use of guidance when designing their research. However, this also highlights a lack of standardisation of ethical approaches in health research with papers citing different or multiple ethical guidelines including those published prior to the last decade or from wider, less relevant disciplines such as marketing or education. This lack of uniform guidance for health research makes it challenging for researchers seeking to design online studies and for ethical review committees seeking to make consistent decisions about how such research should be conducted [41–44]. Previous reviews have also noted the variation in practices across social media research specifically and called for concrete guidelines on research ethics for social media research to be made available [45]. However, it should be noted that there has been an argument against a “one size fits all” approach to online research due to the diversity in online cultures, values, and platforms [46]. Whilst these complexities should not deter researchers from conducting online research they often instead require an individual assessment of the potential ethical issues [47]. However, the reflexive approach taken to online ethics by the BPS [8], BSA [9] and AoIR [11] (termed “ethical pluralism” by the AoIR [11]) and commonality in key themes, suggests that it is possible to develop research guidance which covers varying health research aims and approaches.

Broadly, the guidance documents covered four common thematic areas and recommended 1) decisions about seeking consent to use publicly available online data should balance the scientific value to the research with environmental factors relating to the online community including sensitivity of the research topic, vulnerability of populations and the potential for community disruption 2) there is ambiguity around which online spaces are public and private which can vary according to individual, online community, cultural and legal perceptions 3) researchers must therefore take active steps to protect participants from harm relative to the perceived risk, for example through anonymisation of data, and 4) researchers must also protect themselves from individual and reputational harm by seeking the correct ethical approvals for their research. Overall, there was good coverage of these principles within the selected papers with the principles of anonymity (n = 16) and perceptions of privacy (n = 12) most frequently included. Issues of consent (n = 7) and potential researcher harm (n = 7) were considered less often.

Notably across these themes, the guidance documents recommended decisions were made relative to the sensitivity of the research topic and vulnerability of the population under study. Examples of sensitive research areas included mental health [8], experiences of personal illness [9] and grief and trauma [11]. Vulnerable populations included women, children and young people, people in certain emotional states and LGBTQ+ people [8,11]. From our reading of this guidance, it was clear to us that a significant proportion of research undertaken in health-related disciplines could be considered sensitive by this definition as they involve the sharing of personal health experiences [48] or data, cover sensitive topics (such as mental health, chronic conditions, or substance use) and aim to include or target vulnerable populations [49]. This suggest that health researchers will be required to consider many of the more complex issues within the guidance as well as ethical safeguards around such issues as potential confidentiality breaches, the collection of sensitive data, or the unauthorised reuse of it [50]. Health research has a long- and well-established tradition of ethical guidance. In many countries, research taking place on healthcare premises or with patients is reviewed by separate institutional boards within healthcare organisations and there are many standardised expectations and procedures which govern how health related research can take place. For example, in the UK approval for research with National Health Service (NHS) patients must be granted by an NHS ethical committee and then individual approval provided by each hospital or healthcare trust where research will take place. Given the rigorous ethical requirements for health-related research in many countries, it is therefore quite surprising that guidance for online health research has not been produced by any official health organisations. This guidance would be valuable in assisting health researchers to reflexively design their studies to meet the ethical requirements in both health-related disciplines and the wider online research community.

### Limitations

This scoping review only included studies which were of relevance to health-related researchers. Given the lack of guidance from health organisations, this may have excluded some broader ethics documents which are being used by health researchers to inform their studies. We limited our scoping review to guidance and papers published in the past 10 years to reflect the fast-changing nature of online communities and online research methodologies. It is possible this may have excluded some earlier guidance in health-related fields, although if these documents have not been updated then their relevance to current health related researchers may be limited. Our review also identified some emerging issues which were beyond the scope of this paper including the ethics of digital images [11] and the ethical impacts of big data in health, particularly prospective studies which predict sensitive health issues such as mental health or chronic conditions [27,51] which warrant further research. Similarly, we only included papers which presented some form of recommendation or guidance to other researchers on ethical online research practices. Further research could consider online health studies more generally to understand which key ethical principles are being applied, although the early stage of our review suggests that reporting of ethical practices in online research studies is inconsistent.

## Conclusion

This scoping review aimed to synthesise current best ethical practice for online research in health -related fields. The review identified that there are currently no specific guidelines aimed at health researchers, with the most cited guidance coming from broader methodological perspectives (Franzke et al, 2020) or auxiliary fields [8,9]. As a consequence, many researchers had attempted to synthesise the recommendations from this guidance, their own research and previous studies to produce their own recommendations on ethical practice. Our review identified four key principles of ethical practice which were well cited in the online health research literature 1) a reflexive approach to seeking informed consent 2) protecting the privacy and anonymity of online participants 3) protecting participants from any harm associated with analysing their online data 4) preventing researchers from harm whilst undertaking online research. Across all the existing guidance, ethical decision making was framed in relation to the sensitivity of the research topic and vulnerability of the population. Given that much health research focuses on sensitive topics and populations, our review recommends unifying guidance specific for health researchers to help them design reflexive and ethical online research. Next steps should be focused on developing tailored health research guideline which draws on the experiences of health researchers working in this domain, as well as providing particular consideration to research across sensitive topics and with vulnerable groups.

## Supporting information

S1 Checklist PRISMA 2020 checklist. (DOCX)

S1 File (DOCX)
