# Supplementary material for: Ethical guidance for conducting health research with online communities: A scoping review of existing guidance
Source: PLoS One. 2024 May 17;19(5):e0302924. doi: 10.1371/journal.pone.0302924 (PMC11101025; doi:10.1371/journal.pone.0302924)
Supplement: S1 File — (DOCX) [file pone.0302924.s002.docx]

**Search Strategy**

| Group | Terms |
| --- | --- |
| Online | Online OR internet OR cyber* OR “web-based” OR “web based” OR “social media” OR “social networking” OR Facebook OR forum OR “discussion board” OR Instagram OR Twitter OR YouTube OR snapchat OR TikTok OR Reddit |
| Research | Research* OR method* OR qualitative OR quantitative OR “data scraping” OR “data-scraping” OR analysis OR “data collect*” OR netnography OR ethnography OR “social network analysis” OR “content analysis” OR “textual analysis” OR “thematic analysis” |
| Ethics | Ethic* |
| Policy/Guidance | Policy OR Guid* OR Policies OR Model OR Theor* OR Framework OR Protocol OR Approach OR Strateg* OR Rule OR “How to” |

**Sample Search (Scopus)**

| Scopus  2013-2022 | ( TITLE-ABS-KEY ( online OR internet OR cyber* OR "web-based" OR "web based" OR "social media" OR "social networking" OR facebook OR forum OR "discussion board" OR instagram OR twitter OR youtube OR snapchat OR tiktok OR reddit ) ) AND ( TITLE-ABS-KEY ( research* OR method* OR qualitative OR quantitative OR "data scraping" OR "data-scraping" OR analysis OR study OR design OR project OR "data collect*" OR netnography OR ethnography OR "social network analysis" OR "content analysis" OR "textual analysis" OR "thematic analysis" ) ) AND ( TITLE-ABS-KEY ( policy OR guid* OR policies OR model OR theor* OR framework OR protocol OR instruct* OR approach OR strateg* OR program* OR practice OR rule OR "How to" ) ) AND ( TITLE ( ethic* ) ) | 1891 |
| --- | --- | --- |
